# Reading ability in children with comorbid developmental dyslexia and developmental language disorder: a systematic review: Reading in Children with DD and DLD

**DOI:** 10.1007/s11881-026-00369-y

**Published:** 2026-04-29

**Authors:** Georgia Andreou, Panagiota Sepeti

**Affiliations:** Department of Special Education, University of Thessaly, Volos, Greece

**Keywords:** Developmental dyslexia, Developmental language disorder, Comorbidity, Reading ability

## Abstract

This systematic review aimed to investigate (a) the core characteristics of reading ability in children with comorbid Developmental Dyslexia (DD) and Developmental Language Disorder (DLD), (b) the shared and distinct features of reading performance among children with DD, DLD, and comorbid DD + DLD, and (c) the linguistic and cognitive factors associated with their reading outcomes. Following the Preferred Reporting Items for Systematic Reviews and Meta-Analyses (PRISMA 2020) guidelines, systematic searches were conducted in Web of Science, Scopus, and PubMed between January and May 2025, and a total of 18 empirical studies (published between 2000 and 2023) met the inclusion criteria. Overall, the included studies were conducted across four languages, comprising 15 cross-sectional studies and three longitudinal cohort studies. Risk of bias was assessed using the critical appraisal tools developed by the Joanna Briggs Institute (JBI). Children with comorbid DD and DLD exhibited the most severe and persistent reading difficulties across decoding, fluency, and comprehension tasks, reflecting an additive burden of phonological and language deficits. Comparisons across groups showed that DD primarily affected decoding, DLD impacted comprehension, and the comorbid profile encompassed both types of deficits. Key predictors of poor reading performance were phonological awareness, rapid automatized naming (RAN), semantic knowledge, and syntactic processing. In conclusion, children with comorbid DD and DLD present a qualitatively distinct reading profile that current diagnostic systems may fail to capture, and early identification as well as interventions targeting core language skills, particularly phonological abilities, are essential, while future research should prioritize longitudinal designs, cross-linguistic validation, and diagnostic tools that account for comorbidity.

**Supplementary Information:**

The online version contains supplementary material available at https://doi.org/10.1007/s11881-026-00369-y.

## Introduction

Reading ability is one of the most crucial skills a child develops at the beginning of schooling, as it is closely linked to academic success, everyday functionality, and overall cognitive development (Mody, 2003; Papadimitriou et al., 2017; Snowling et al., 2020; Tallal, 2003). As a complex skill, reading involves both word decoding and text comprehension, processes that rely on phonological processing as well as linguistic and semantic abilities (Catts et al., 2005; Hjetland et al., 2017). Reading comprehension, however, is not an independent component but emerges from the dynamic interaction between decoding and language comprehension processes. In this sense, oral language is not only a developmental foundation but also an active contributor to the construction of meaning from text. In the present review, the term “reading ability” is used as a broad construct encompassing three core components of reading: word-level decoding, reading fluency, and reading comprehension. This conceptualization is consistent with the Simple View of Reading (Catts et al., 2005; Gough & Tunmer, 1986; Hoover & Gough, 1990), which posits that reading comprehension arises from the interaction between decoding and language comprehension processes. Within this theoretical framework, it is well documented that children with a history of language difficulties are at increased risk for developing reading deficits, which may lead to educational disadvantage and exclusion (Bishop & Snowling, 2004; Catts et al., 2005; Stavrakaki, 2001; Talli et al., 2016). Within this framework, distinct developmental profiles can be understood as reflecting impairments in different components of reading, with dyslexia primarily associated with decoding difficulties and DLD with language comprehension difficulties.

A condition frequently linked to such reading deficits is Developmental Dyslexia (DD), a prevalent neurodevelopmental disorder with a persistent developmental trajectory, classified as a specific learning disability (Lyon et al., 2003; Snowling, 2008). Developmental dyslexia is characterized by persistent difficulties in accurate and/or fluent word recognition and spelling, which are not attributable to inadequate instruction or sensory impairments (Lyon et al., 2003; Melby-Lervåg et al., 2012; Nation et al., 2004; Ramus et al., 2003; Snowling, 2008). Evidence indicates that genetic heritability represents a substantial risk factor for its occurrence (Snowling & Melby-Lervåg, 2016). Children with DD may experience difficulties in phonological awareness, rapid automatized naming, and phonological memory (Hulme et al., 2015; Ramus et al., 2013). However, not all individuals with dyslexia exhibit the same pattern of cognitive-linguistic difficulties, reflecting the heterogeneity of the disorder. These difficulties are primarily associated with word-level decoding and may also impact reading comprehension, particularly in the presence of co-occurring language weaknesses.

Another prevalent neurodevelopmental disorder closely related to reading development is Developmental Language Disorder (DLD). According to the international consensus framework CATALISE (Criteria and Terminology Applied to Language Impairments: Synthesising the Evidence) (Bishop et al., 2017), the term DLD is used to describe persistent difficulties in oral language that have a substantial impact on functional communication and academic achievement, in the absence of a known biomedical etiology, such as neurological, genetic, or sensory conditions, and constitutes a subtype of the broader category of Language Disorder. Within this framework, the term Specific Language Impairment (SLI), which was widely used in earlier literature, is now considered a subset of DLD, as all children meeting criteria for SLI fall within the definition of DLD, whereas the reverse does not apply (Bishop et al., 2017). More specifically, DLD encompasses a heterogeneous range of language profiles and does not require a discrepancy between verbal and nonverbal abilities, as it includes children across a broader range of nonverbal cognitive abilities (typically within two standard deviations of the mean), thereby encompassing but not being limited to cases previously described as SLI, nor does it exclude the co-occurrence of other neurodevelopmental disorders (Bishop et al., 2017). DLD manifests as impairments in one or more language domains, including semantics, morphology, and syntax, while phonological and pragmatic difficulties may also be present but are often associated with comorbidity with other neurodevelopmental disorders rather than core features of DLD (Andreou et al., 2023; Bishop & Snowling, 2004; Brizzolara et al., 2006; Stavrakaki, 2001). These language difficulties significantly affect reading development, particularly reading comprehension, as children rely on language skills to construct meaning from text through the formation of coherent mental representations, and limitations in these skills restrict access to word meaning and sentence structure (Catts et al., 2005; Nation et al., 2004). Although some children with DLD may develop adequate decoding skills, their limited language foundation often undermines their ability to effectively organize and interpret written language (Bishop & Snowling, 2004), and these difficulties are typically persistent, with potential long-term consequences for academic, cognitive, and educational development (Diamanti et al., 2018; Erisman & Blom, 2020; Spanoudis et al., 2019).

Developmental Dyslexia (DD) and Developmental Language Disorder (DLD) are recognized as distinct neurodevelopmental conditions (Bishop et al., 2009; Catts et al., 2005), however comorbidity between them is common, with many children exhibiting features of both disorders. Population-based studies indicate that comorbidity between DD and DLD is observed in a substantial proportion of affected children, with approximately one third of children with language disorder meeting broader criteria for dyslexia in later school years, while prevalence estimates range between 17% and 29% when more conservative, IQ-referenced definitions are applied (Catts et al., 2005). In the present review, comorbidity is defined as the co-occurrence of two distinct neurodevelopmental disorders within the same individual, without implying diagnostic overlap or shared etiology (Bishop & Snowling, 2004; Catts et al., 2005). This comorbidity has been associated with more severe and complex reading difficulties, likely due to the co-occurrence of phonological and linguistic difficulties, reflecting broader and more complex learning profiles rather than generalized cognitive deficits (Bishop et al., 2009; Kelso et al., 2007; Ramus et al., 2013; Snowling et al., 2020). This pattern can be interpreted within the framework of the Fully Distinct Hypothesis, which posits that DD and DLD arise from partially independent developmental mechanisms and that comorbidity reflects the co-occurrence of two distinct disorders rather than a single underlying condition or a severity continuum (Bishop & Snowling, 2004; Catts et al., 2005). This distinction is consistent with the Simple View of Reading, as it reflects impairments in different components of reading. Despite their frequent co-occurrence, DD and DLD are differentiated diagnostically based on the primary domain of impairment, with DD primarily characterized by word-level reading and spelling difficulties, and DLD by broader oral language deficits (Bishop & Snowling, 2004; Catts et al., 2005). Nevertheless, much of the existing literature has focused on comparisons between DD and DLD as independent groups, without systematically investigating children who meet criteria for both. Studies such as those by Rispens and Been (2007), Lauterbach et al. (2017), and Guimarães and Mousinho (2021) offer valuable insights into the distinct profiles of each disorder, but omit examination of the comorbid population. Rather than reflecting a lack of research on this subgroup, this may be due to differences in terminology, as earlier studies often used related constructs (e.g., SLI or LI) that may have included children who meet current criteria for DLD. This highlights an important direction for future research, particularly given the potential complexity of their reading profiles. Gaining a better understanding of their differentiated profiles is essential for the development of accurate diagnostic tools and tailored intervention approaches.

Given the above, the aim of the present systematic review is to investigate the reading ability of children with comorbid DD and DLD. Specifically, the review seeks to (a) systematically describe their reading performance profiles, (b) compare their reading outcomes with those of children diagnosed solely with DD or DLD, and (c) identify the linguistic and cognitive factors associated with their reading abilities.

## Objectives

The present review is guided by the following research questions:

1. What are the core characteristics of reading ability in children with comorbid Developmental Dyslexia (DD) and Developmental Language Disorder (DLD)?
2. What are the shared and distinct features of reading performance among children with DD, DLD, and comorbid DD and DLD?
3. Which linguistic and cognitive factors are associated with reading performance in children with comorbid DD and DLD?

### Method

#### Review framework and approach

This systematic review followed the PRISMA 2020 (Preferred Reporting Items for Systematic Reviews and Meta-Analyses) guidelines to ensure methodological rigor, transparency, and replicability (Page et al., 2021). The completed PRISMA 2020 checklist is provided as Supplementary Material. Empirical studies focusing on the reading abilities of children with comorbid Developmental Dyslexia (DD) and Developmental Language Disorder (DLD) were identified through structured searches in three major scientific databases: Web of Science, Scopus, and PubMed. In addition, the review was preregistered on the Open Science Framework (OSF; link blinded for peer review) to enhance transparency and ensure reproducibility. The screening of titles and abstracts, as well as the full-text eligibility assessment, was conducted by a single reviewer, using predefined inclusion and exclusion criteria in accordance with PRISMA 2020 guidelines. Decisions at each stage were guided by explicit eligibility criteria to ensure consistency and transparency throughout the selection process.

## Inclusion and exclusion criteria

Eligible studies met predefined inclusion and exclusion criteria. Specifically, original empirical studies published in English or Greek between 2000 and 2025 were included if they examined children aged 3 to 18 years. Studies had to focus on populations with comorbid Developmental Dyslexia (DD) and Developmental Language Disorder (DLD), with both conditions clearly diagnosed based on the criteria applied in each primary study. Studies were included only if diagnostic procedures were adequately described and based on standardized assessments or clearly defined and widely accepted diagnostic criteria. Studies that did not provide sufficient information regarding diagnostic procedures or criteria were excluded. Studies comparing this comorbid group to children with DD-only, DLD-only, or typically developing peers were also eligible.

For the purposes of the present systematic review, Developmental Dyslexia (DD) was considered clearly defined when studies reported persistent difficulties in the accuracy and/or fluency of word reading, as assessed through standardized and validated reading measures, in the absence of sensory impairments, insufficient educational experience, or nonverbal cognitive impairment. Developmental Language Disorder (DLD) was considered clearly defined when studies documented significant and persistent difficulties in oral language affecting functional communication, as assessed through standardized language measures or formal clinical diagnosis, in the absence of a known biomedical etiology. Only studies in which the diagnostic criteria for DD and DLD were explicitly described with sufficient clarity, in accordance with established research or clinical standards, were included. Consequently, in the present systematic review, studies that employed earlier terminology (e.g., Specific Language Impairment) were retained and reclassified under the DLD framework when their diagnostic descriptions were consistent with the CATALISE consensus definition (Bishop et al., 2017). Studies were included only when they provided clear and explicit diagnostic criteria for language impairment and sufficient information on how this was assessed. No additional cut-off thresholds (e.g., standard deviation criteria) or specific nonverbal IQ (NVIQ) ranges were imposed beyond those reported in each primary study.

With regard to the operational use of the term Developmental Language Disorder (DLD), the present review did not impose post hoc uniform diagnostic criteria or cut-off thresholds related to nonverbal intelligence or severity of language impairment. Instead, the diagnostic definitions and eligibility criteria applied in each primary study were retained. This decision reflects the methodological reality of the literature, as a substantial proportion of available studies predates the CATALISE consensus and relies on earlier diagnostic labels, such as Specific Language Impairment (SLI), often accompanied by more restrictive nonverbal IQ criteria. Consequently, the present review does not intentionally restrict the DLD construct to a specific range of nonverbal intelligence, but rather synthesizes the available evidence as it is represented in the literature. In addition, both Developmental Dyslexia (DD) and Developmental Language Disorder (DLD) were accepted on the basis of either formal clinical diagnoses or clearly specified research-based cut points, provided that diagnostic procedures were adequately described. Variability in diagnostic thresholds across studies was not treated as an exclusion criterion, but is acknowledged as a source of heterogeneity and addressed in the limitations of the review.

With regard to literacy-related abilities, studies were accepted if they included either direct measures (e.g., decoding, word recognition, reading comprehension, spelling) or indirect indicators (e.g., phonological awareness, phonological memory, rapid automatized naming).

Studies were excluded if they: (a) involved adults or participants without clearly documented comorbidity of DD and DLD; (b) failed to provide sufficient description of the sample or the assessment procedures used; or (c) were theoretical articles, reviews, dissertations, conference abstracts, or other non-empirical publications.

## Search strategy

Searches were conducted between January and May 2025 across three major academic databases: Web of Science, Scopus, and PubMed. Each search followed a structured protocol based on predefined thematic categories, namely reading ability, developmental dyslexia, developmental language disorder, and age group. The search terms were selected to capture the broad range of terminologies used in the literature, and conceptually equivalent terms were employed to encompass alternative labels such as reading disability, language impairment, and related subtypes (e.g., specific word-reading disability), through the use of broader terms including reading disorder, decoding, word recognition, and language impairment.

In Web of Science, the search was implemented using the “Topic (TS=)” field in Advanced Search mode. The terms used included: “reading skills” OR “reading ability” OR “reading comprehension” OR “reading difficulties” OR “reading development” OR “reading performance” OR “decoding” OR “word recognition” OR “phonological processing” OR “literacy development”, combined with “dyslexia” OR “developmental dyslexia” OR “reading disorder”, and “language disorder” OR “developmental language disorder” OR “language impairment” OR “specific language impairment” OR “SLI” OR “oral language”. For the age range, terms such as child, children, school-age, school-aged, students, and preschool were used. Filters were applied for publication year (2000–2025), language (English or Greek), and document type (Articles). This search yielded 437 records.

In Scopus, a similar search strategy was applied using the “Article Title, Abstract, Keywords” fields. The same search terms and filters were used, limiting results to peer-reviewed articles in English or Greek, published between 2000 and 2025. A total of 296 articles were identified.

In PubMed, the search was conducted in the “Title/Abstract” fields using the same terms and filtering criteria. This search returned 106 potentially relevant records.

Overall, the combined database search yielded 839 articles prior to duplicate removal and screening. See Table 1 for an overview of included studies.

## Study selection process

The initial search across the three databases (Web of Science: 437, Scopus: 296, PubMed: 106) yielded a total of 839 articles. During the initial title screening, 553 articles were excluded due to irrelevance. The remaining 286 articles were imported into Mendeley for reference management and duplicate removal. After removing 63 duplicates, 223 unique records remained for abstract screening.

**Fig. 1:**
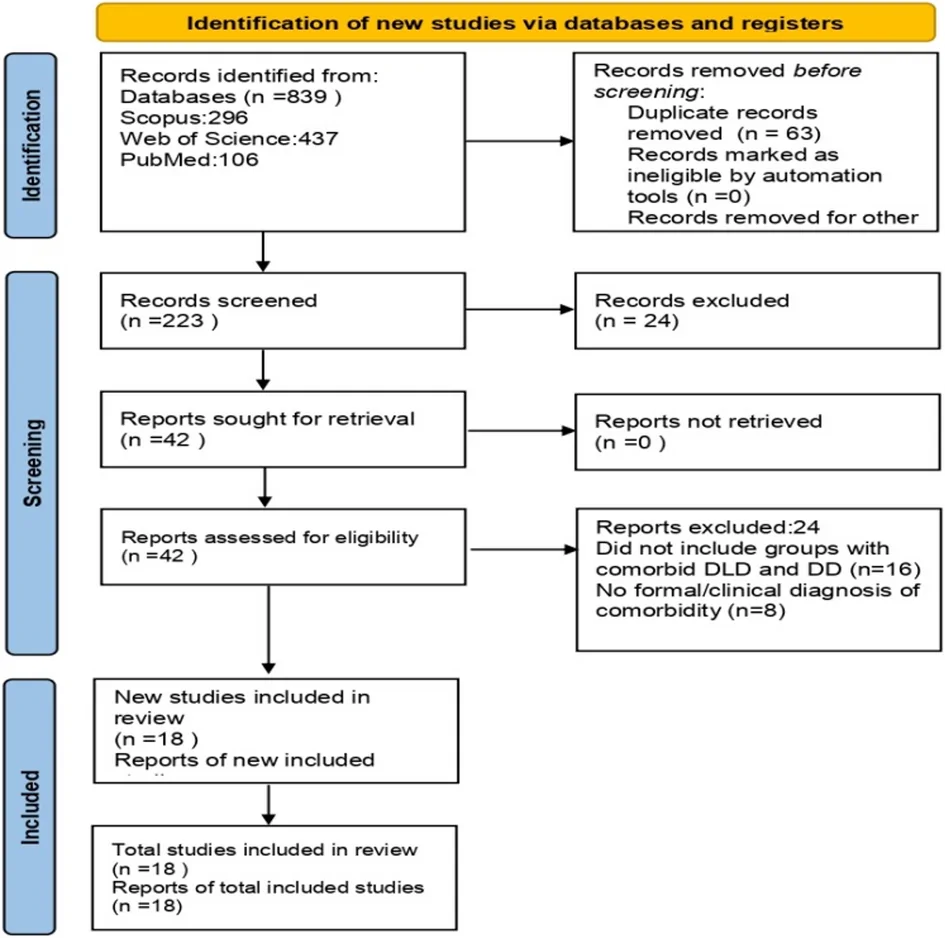
PRISMA flow diagram of the study selection process

Following the abstract review, 181 articles were excluded because they did not meet the inclusion criteria (e.g., studies focusing solely on DD or DLD without comorbidity). The remaining 42 articles underwent full-text screening. Of these, 16 were excluded due to the absence of a clearly defined comorbid DD and DLD group, and 8 due to insufficient diagnostic information (e.g., lack of clinical or standardized criteria). Ultimately, 18 studies met all the inclusion criteria set for this study and were included in the systematic review. A summary of the identification and screening process is presented in Fig. 1.

### Risk of bias assessment

Risk of bias was assessed using the critical appraisal tools developed by the Joanna Briggs Institute (JBI; Joanna Briggs Institute 2020a, b). Specifically, for the three studies employing a longitudinal cohort design, the JBI checklist for cohort studies was used, whereas the checklist for analytical cross-sectional studies was applied to the remaining 15 cross-sectional studies. The use of distinct tools was deemed necessary to adequately reflect the methodological design of each study and ensure the validity of the appraisal.

Each tool consists of a series of criteria (ranging from 8 to 11 items) that evaluate methodological rigor, including sample description, clarity of inclusion criteria, reliability of measurements, and management of potential confounding factors. For each item, responses were recorded as “Yes,” “No,” “Unclear,” or “Not applicable.” The appraisal was conducted by a single reviewer based on information provided in the full text of each article. See Table 1 for the cross-sectional studies and Table 2 for the cohort studies.

**Table 1:** Critical appraisal of included analytical cross-sectional studies using the JBI

Study	Q1	Q2	Q3	Q4	Q5	Q6	Q7	Q8
Eisenmajer et al. (2005)	Yes	Yes	Yes	Yes	Unclear	No	Yes	Yes
Scuccimarra et al. (2008)	Yes	Yes	Yes	Yes	Unclear	No	Yes	Yes
Fraser et al. (2010)	Yes	Yes	Yes	Yes	Unclear	No	Yes	Yes
Wong et al. (2010)	Yes	Yes	Yes	Yes	Unclear	No	Yes	Yes
McArthur and Castles (2011)	Yes	Yes	Yes	Yes	Unclear	No	Yes	Yes
McCarthy et al. (2012)	Yes	Yes	Yes	Yes	Unclear	No	Yes	Yes
Kim and Lombardino (2013)	Yes	Yes	Yes	Yes	No	Unclear	Yes	Yes
Wong et al. (2015)	Yes	Yes	Unclear	Yes	Unclear	Yes	Yes	Unclear
Loucas et al. (2016)	Yes	Yes	Yes	Yes	Unclear	Yes	Yes	Yes
Wong et al. (2017)	Yes	Yes	Yes	Yes	Unclear	Yes	Yes	Yes
Mengisidou and Marshall (2019)	Yes	Yes	Yes	Yes	Yes	Yes	Yes	Yes
Spanoudis et al. (2019)	Yes	Yes	Unclear	Unclear	Yes	Yes	Unclear	Yes
Mengisidou et al. (2020)	Yes	Yes	Yes	Yes	Unclear	Unclear	Yes	Yes
Snowling et al. (2020)	Yes	Yes	Yes	Yes	Unclear	No	Yes	Yes
Ehrhorn et al. (2021)	Yes	Yes	Yes	Yes	Unclear	Unclear	Yes	Yes

Note. Response codes: Yes – Criterion clearly met No – Criterion clearly not met Unclear – Reporting insufficient to permit judgement. Checklist items: Q1: Criteria for inclusion in the sample clearly defined Q2: Study subjects and setting described in sufficient detail Q3:Exposure measured in a valid and reliable way Q4: Objective, standard criteria used for condition measurement Q5: Confounding factors identified Q6: Strategies to deal with confounding factors stated Q7: Outcomes measured in a valid and reliable way Q8: Appropriate statistical analysis used

**Table 2:** Critical appraisal of included cohort studies using the JBI checklist for cohort studies

Study	Q1	Q2	Q3	Q4	Q5	Q6	Q7	Q8	Q9	Q10	Q11
Catts et al. (2005)	Yes	Yes	Yes	Unclear	Unclear	Yes	Yes	Yes	Yes	Unclear	Yes
Snowling et al. (2019)	Yes	Yes	Yes	Yes	Yes	Yes	Yes	Yes	Unclear	Unclear	Yes
Duff et al. (2022)	Yes	Yes	Yes	Yes	Yes	Yes	Yes	Yes	Unclear	Unclear	Yes

Note.Q1: Groups similar and recruited from same population Q2: Exposures measured similarly for both groups Q3: Exposure measured in a valid and reliable way Q4: Confounding factors identified Q5: Strategies to deal with confounding stated Q6: Participants free of outcome at baseline Q7: Outcomes measured in a valid and reliable way Q8: Follow-up time sufficient Q9: Follow-up complete, reasons for loss described Q10: Strategies to address incomplete follow-up used Q11: Appropriate statistical analysis used

## Results

A total of 18 studies met the inclusion criteria and were included in the present systematic review. These studies were published between 2000 and 2023. The majority followed a cross-sectional design, while three studies were longitudinal cohort studies. The age range of participants across studies spanned from 3.5 to 17 years. All studies included children with comorbid Developmental Dyslexia (DD) and Developmental Language Disorder (DLD). Comorbidity was established either through official clinical diagnoses by professionals or through documented performance-based criteria, such as low scores on standardized language or cognitive assessments. The reading measures used varied across studies, but typically included word and nonword decoding, reading fluency and speed, reading comprehension, phonological processing, and rapid automatized naming. See Table 3 for an overview of included studies.

### Reading profile of children with comorbid DD and DLD

Of the studies included in the present systematic review, six specifically focused on examining the reading profile of children with comorbid Developmental Dyslexia (DD) and Developmental Language Disorder (DLD). These studies investigated decoding, fluency, and comprehension skills, as well as cognitive and linguistic factors associated with reading performance.

Catts et al. (2005) examined the reading development of children with Specific Language Impairment (SLI), dyslexia, and comorbid SLI and dyslexia, aiming to clarify the impact of comorbidity on reading ability. In a longitudinal sample of 527 children assessed from kindergarten to eighth grade, reading ability was measured using the Word Identification and Word Attack subtests of the Woodcock Reading Mastery Tests. Language skills had been evaluated earlier in preschool with the Test of Language Development–Primary, Second Edition (TOLD–2:P) and a narrative task.

In the second part of the study, a subsample of 247 children was analyzed, divided into four groups: SLI only (*n* = 43), dyslexia only (*n* = 21), comorbid SLI and dyslexia (*n* = 18), and typically developing children (*n* = 165). Children with comorbidity demonstrated significantly lower performance in both word recognition and phonological processing compared to the single-disorder groups. These findings confirmed that the coexistence of SLI and dyslexia entails an additive burden on decoding and language processing, highlighting that comorbidity is associated with particularly adverse reading profiles.

In addition, Wong et al. (2015) investigated the relationship between reading ability and cognitive skills, including phonological awareness, phonological memory, and rapid automatized naming (RAN). The study involved 94 children aged 6.7 to 7.11 years, who were assigned to four groups: dyslexia only (*n* = 10), SLI only (*n* = 19), comorbid dyslexia and SLI (*n* = 25), and typically developing children (*n* = 40). Reading ability was assessed using the Hong Kong Cantonese Oral Language Assessment Scale (HKCOLAS) and the Hong Kong Test of Specific Learning Difficulties in Reading and Writing, Second Edition (HKT-P[II]), which measure word reading skills and reading speed. Results indicated that children with dyslexia, as well as those with comorbidity, performed significantly lower on the reading measures compared to the SLI-only and typically developing groups. These findings underscore the distinctive role of dyslexia in shaping reading outcomes, suggesting that phonological deficits linked to dyslexia may be the main driver of reading difficulties in the comorbid population.

Furthermore, the longitudinal study by Snowling et al. (2019) investigated the reading development of 234 children aged 3.5 to 8 years, based on early risk indicators for dyslexia and/or language disorder. Participants were divided into four groups: typically developing children (*n* = 73); children with a family history of dyslexia but no language disorder (*n* = 86); children with early language disorder but no family history (*n* = 30); and children with both family history and early language disorder (*n* = 30). At age 8, reading ability was assessed using the Single Word Reading Test (SWRT), the York Assessment of Reading for Comprehension (YARC), and Rapid Automatized Naming (RAN). Children with comorbidity displayed the lowest performance in both decoding and comprehension tasks, highlighting a cumulative effect of language deficits and familial risk. The combination of early language difficulties and family history appeared to be a strong predictor of later reading challenges.

Similarly, the study by Mengisidou and Marshall (2019) focused on 149 monolingual Greek-speaking children aged 6 to 12 years, including 66 children diagnosed with Developmental Dyslexia and Developmental Language Disorder (DDLD) and 83 typically developing peers. Reading skills were assessed using the Greek adaptations of the L’Alouette test, the Reading Test Alpha, and the Diagnostic Test of Reading Difficulties (Porpodas, 2007). These assessments were individually administered in sessions lasting approximately 90 min. Children with DDLD showed significantly lower performance in reading fluency and accuracy, with more pronounced difficulties in nonword reading, indicating limited phonological processing and reduced lexical access.

Extending these findings, the study by Mengisidou et al. (2020) assessed semantic ability and its relationship with reading performance in a sample of 149 children (66 with DDLD and 83 typically developing). Depending on grade level, reading performance was assessed using the Alouette, Reading Test Alpha, and the Diagnostic Test of Reading Difficulties (DIRD). Additionally, word production was examined through a semantic fluency task. Children with DDLD performed significantly lower across all reading measures, particularly in nonword reading, and showed reduced lexical retrieval ability. These findings support the link between semantic structure and effective reading processing.

Likewise, the longitudinal study by Duff et al. (2023) investigated reading development in students from Grade 2 to Grade 4, focusing on children with dyslexia, developmental language disorder (DLD), comorbidity of both conditions, and typical development. Classification of the 448 participants into the four diagnostic groups was conducted in Grade 2 based on composite scores from the Clinical Evaluation of Language Fundamentals–Fourth Edition **(**CELF-4; language skills) and the Woodcock Reading Mastery Tests–Third Edition (WRMT-III; reading decoding). The groups consisted of: dyslexia only (*n* = 45), DLD only (*n* = 91), comorbid dyslexia and DLD (*n* = 78), and typically developing peers (*n* = 234). Reading ability was measured through the Measures of Academic Progress–Reading (MAP–Reading), a computer-adaptive test (CAT) that yields a Rasch Unit (RIT) score. The interpretation of the RIT score varies depending on administration conditions: when test items are read aloud to students, performance primarily reflects language comprehension, whereas when students read independently, performance reflects the combined contribution of decoding and language comprehension. In the present study, students read the test items independently, and therefore MAP–Reading performance reflects the combined contribution of decoding and language comprehension. The results indicated that children with comorbid dyslexia and DLD demonstrated significantly lower reading performance at all time points. While children with only DLD showed similar initial performance to the dyslexia group, they exhibited slower reading progress over time.

### Comparison between DD, DLD, and comorbidity

This review identified seven studies that compared the reading performance of children with Developmental Dyslexia (DD), Developmental Language Disorder (DLD), and comorbid DD and DLD, in order to determine whether the co-occurrence of both disorders results in more severe reading difficulties than either condition alone.

The study by Eisenmajer et al. (2005) examined the reading abilities of 151 children aged 7–12 years with learning difficulties, divided into five groups: DLD (*n* = 25), dyslexia (*n* = 21), comorbid DLD and dyslexia (*n* = 57), general learning disability (*n* = 20), and typically developing peers (*n* = 28). Reading performance was assessed using the Wechsler Individual Achievement Test – Reading subtest (WIAT), while the Clinical Evaluation of Language Fundamentals–Revised/Third Edition (CELF-R/3) was used to classify participants into diagnostic groups. Results indicated that children with comorbid DLD and dyslexia performed significantly lower in decoding tasks compared to both the single-disorder groups and the typically developing group.

In the study by Wong et al. (2010), 39 Cantonese-speaking children aged 6–11 years were assigned to four groups: children with SLI without dyslexia (*n* = 7), children with both SLI and dyslexia (*n* = 13), children with a history of SLI (*n* = 10), and typically developing (TD) children (*n* = 9). Reading ability was assessed using the Chinese Word Reading and One-minute Word Reading subtests of the HKT-P[II]. Children in the comorbid group exhibited the greatest difficulties in both reading speed and accuracy. They also performed poorly on tasks of morphological analysis and showed limited phonological representations, suggesting complex phonological and morphological deficits.

In line with previous evidence, the study by Fraser et al. (2010) included 64 children aged 9–11 years, divided into four groups: children with DLD (*n* = 16), children with Specific Reading Disability (SRD), (*n* = 14), children with comorbid SLI and SRD (*n* = 21), and TD children (*n* = 13). Reading performance was assessed using the British Ability Scales–Second Edition (BAS-II; Single Word Reading) and the Test of Word Reading Efficiency (TOWRE; Sight Word Efficiency), and Phonemic Decoding Efficiency, which measure the accuracy and speed of reading real and pseudowords. The comorbid SLI/SRD group demonstrated the lowest performance overall. Notably, phonological awareness emerged as the strongest predictor of reading performance across all groups.

Ιn addition, Kim and Lombardino (2013) examined 91 children aged 8–12 years, divided into two groups: children with specific reading disability (SRD, *n* = 60) and children with comorbid language impairment and reading disability (LIRD, *n* = 31). Reading skills were assessed using the Gray Oral Reading Test – Fourth Edition (GORT-IV), which measures reading fluency and comprehension, and the RAN subtest of the Comprehensive Test of Phonological Processing (CTOPP). The LIRD group scored significantly lower in both comprehension and verbal fluency. Naming speed was positively correlated with reading fluency in both groups, while only the LIRD group showed a predictive relationship between verbal skills and reading comprehension.

Wong et al. (2017) investigated text comprehension in 82 children aged 7.8–9.5 years, assigned to four groups: typically developing (TD, *n* = 34), DLD only (*n* = 18), DD only (*n* = 8), and comorbid DLD + DD (*n* = 22). Reading ability was assessed using the HKT-P[II], while comprehension was measured using experimental tasks of comprehension monitoring and inferencing. The comorbid group demonstrated the lowest comprehension scores, although they did not differ clearly from the single-disorder groups across all cognitive indicators. Working memory and higher-level language skills accounted for a significant portion of reading performance.

Additionally, Spanoudis et al. (2019) examined 52 children divided into four groups: RD only (*n* = 10), SLI only (*n* = 13), comorbid RD and SLI (COM; *n* = 9), and typically developing (TD) children (*n* = 20). Reading performance was evaluated through decoding tasks (Word Identification, Word Attack, and Word Chains) and comprehension measures (Curriculum-Based Measurement Maze [CBM-Maze] and Test-A). Children in the COM group showed the lowest scores across both decoding and comprehension domains, while reading accuracy and speed were more strongly associated with phonological processing in the single-disorder groups. These findings reinforce the notion that comorbidity exacerbates the severity of reading difficulties.

Finally, the study by Snowling et al. (2020) examined 234 children aged 8–9 years, who were assigned to four groups: children with Developmental Dyslexia (DD, *n* = 21), children with developmental language disorder (DLD, *n* = 38), children with comorbid DD and DLD (*n* = 29), and typically developing children (*n* = 64). In addition, 82 children identified in preschool as being at risk due to early language or literacy difficulties but who later showed typical outcomes were included as an “at-risk with typical outcome” group. Reading ability was assessed through standardized measures of decoding, phonological processing, and reading comprehension, including the York Assessment of Reading for Comprehension (YARC), the Test of Word Reading Efficiency (TOWRE), and subtests from the Clinical Evaluation of Language Fundamentals–Fourth Edition (CELF-4). Children with comorbid DD and DLD demonstrated the lowest performance in reading comprehension, supporting the hypothesis of an additive effect of phonological and language deficits on reading comprehension outcomes.

Overall, the strength and weight of the evidence should be interpreted with caution. While some findings are supported by studies with larger samples and clearer diagnostic differentiation between Developmental Dyslexia, Developmental Language Disorder, and comorbid profiles, a substantial proportion of the available evidence is derived from small-scale, cross-sectional studies. In addition, variability in diagnostic criteria and heterogeneity in the assessment of reading-related outcomes (e.g., decoding, fluency, and comprehension) limit the comparability of findings across studies and reduce the strength of conclusions regarding differentiated reading profiles in comorbid populations.

### Underlying mechanisms and predictors of reading ability

This review identified five studies that examined in depth the underlying mechanisms and predictors of reading ability in children with comorbid Developmental Dyslexia (DD) and Developmental Language Disorder (DLD). These studies focused primarily on phonological skills, language abilities, and cognitive markers, aiming to clarify their contribution to the reading profile associated with comorbidity.

The study by Scuccimarra et al. (2008) was conducted with 60 Italian-speaking children divided into three groups: 15 children with developmental dyslexia and a history of language impairment (SLI group), 15 children with dyslexia but no indication of language difficulties (non-SLI group), and 30 typically developing children (control group). Reading ability was assessed using the MT Reading Test (standardized Italian test for oral reading speed and accuracy), along with tasks involving word and pseudoword recognition at various phonological levels. Children with dyslexia, regardless of the presence of SLI, showed equally poor performance in decoding tasks, suggesting that comorbid language impairment did not impose additional reading difficulties during the early school years.

Further exploring phonological mechanisms, the study by McArthur and Castles (2011) included 73 children aged 7–12 years, assigned to four groups: SRD (*n* = 42), SLI (*n* = 5), SRD + SLI (*n* = 18), and typically developing (TD) children (*n* = 8). The procedure involved an individual session with five phonological processing tasks: phoneme discrimination, phonological awareness, rapid automatized naming (RAN), phonological short-term memory, and quality of phonological representations. Children with comorbidity exhibited broader and more severe phonological deficits compared to the other groups, while children with SLI-only did not show significant phonological difficulties.

Moreover, in the study by McCarthy et al. (2012), 129 fourth-grade children were classified into four groups: children with specific language impairment (SLI, *n* = 43), developmental dyslexia (DD, *n* = 21), comorbid SLI and DD (SLI + DD, *n* = 18), and typically developing (TD) children (*n* = 47). Reading performance was assessed using the Word Identification and Word Attack subtests of the Woodcock Reading Mastery Test – Revised. Diagnostic classification was based on criteria established in preschool as part of a longitudinal epidemiological study. Children in the comorbid group demonstrated significantly lower decoding performance compared to the single-disorder and TD groups, confirming the additive burden associated with comorbidity. Notably, decoding ability was more discriminative among the groups than oral language proficiency.

Additionally, the study by Loucas et al. (2016) examined 64 children aged 5–17 years, divided into three groups: children without specific language impairment (SLI) or reading decoding impairment (RDI; *n* = 18), children with SLI but no RDI (*n* = 20), and children with comorbid SLI and RDI (*n* = 22). Reading ability was assessed using the Test of Word Reading Efficiency (TOWRE), which includes both real and pseudoword reading tasks. Phonological processing was evaluated through the Children’s Nonword Repetition test (CNRep), Nonword Discrimination (NWD), and the Comprehensive Test of Phonological Processing (CTOPP). Language was assessed using the Clinical Evaluation of Language Fundamentals (CELF-3/4), while nonverbal intelligence was measured with the Wechsler Intelligence Scale for Children – Third Edition (WISC-III). Children with both SLI and RDI showed more severe phonological processing deficits, especially in complex tasks, which the authors attributed to a functional impairment in the phonological output system.

Finally, the study by Ehrhorn et al.(2021) examined phonological processing in 150 children aged 7–9 years diagnosed with DD (*n* = 20), DLD (*n* = 40), comorbid DD and DLD (*n* = 41), and TD children (*n* = 59). Group classification was based on participants’ performance on the Woodcock Reading Mastery Test – Third Edition (WRMT-3) for reading skills and the Clinical Evaluation of Language Fundamentals – Fourth Edition (CELF-4) for language abilities. Phonological processing was assessed using two versions of a nonword repetition task: the standardized subtest from the Comprehensive Test of Phonological Processing – Second Edition (CTOPP-2) and an experimental version incorporating auditory and audiovisual stimuli. Children in the comorbid group demonstrated significantly lower performance on both task formats compared to the single-disorder groups, indicating a cumulative phonological deficit.

**Table 3:** Summary of included studies

No	Authors (Year)	Country	Sample (Age)	Comparison Groups	Assessment Tools (Reading & Language)	Reading Variables	Findings on Reading Ability
1	Catts et al. (2005)	USA	Ν = 527, Ages 5–14	SLI only DD only SLI + Dyslexia Typically Developing	Woodcock Reading Mastery Tests (Word Identification, Word Attack), TOLD-2:P, narrative production task	Word recognition accuracy, phonological processing, reading comprehension	Children with comorbid SLI and DD exhibited the most severe reading and phonological impairments. The SLI-only group showed adequate word recognition but mild phonological deficits.
2	Eisenmajer et al. (2005)	Australia	*N* = 151, Ages 7–12	SRD, SLI, LIRD, GD, ND	WIAT (Reading subtest), CELF-R / CELF-3 (language assessment)	Single-word reading accuracy	LIRD group showed significantly lower word reading scores than SRD and SLI. Over half of the sample had both impairments, indicating that comorbidity leads to more severe reading difficulties.
3	Scuccimarra et al. (2008)	Italy	*N* = 60, Ages 7–8	DD + SLI, DD-only, TD	MT Reading Test (words and nonwords; measures of speed and accuracy)	Reading speed, accuracy, error types in word and nonword reading	Both DD + SLI and DD-only groups showed similar deficits in reading speed and accuracy, with significantly more errors than typically developing peers. No significant differences were found between the two dyslexic groups.
4	Fraser et al. (2010)	UK	*N* = 64, Ages 9–11	SLI, SRD, SLI + SRD, TD/(CA)	BAS-II (single word reading), TOWRE (sight word efficiency, phonological decoding efficiency)	Word recognition (timed & untimed), decoding (real & pseudowords), reading speed & accuracy	Children with SRD and SLI + SRD had significant deficits in all reading measures compared to the TD group. The SLI + SRD group showed the most severe impairments. The SLI-only group performed similarly to TD peers on timed tasks but worse on untimed reading.
5	Wong et al. (2010)	Hong Kong	*N* = 39, Ages 6-11.3	SLI-only, SLI + D (Comorbid), SLI-History (resolved DLD), TD	HKT-P[II]: Chinese Word Reading (accuracy), One-minute Word Reading (timed fluency – number of words read in 1 min)	Word reading accuracy; Word reading fluency/speed	The SLI + D group demonstrated significantly lower performance in both reading accuracy and fluency compared to all other groups. The SLI-only group performed better in reading, despite language deficits. Reading was the domain with the most pronounced differences between comorbid and non-comorbid groups. The presence of dyslexia led to additive negative effects on reading skills.
6	McArthur and Castles (2011)	Australia	*N* = 73, Ages 7–12	SRD only, SLI only, SRD + SLI Control	Phonological processing tasks: phoneme representation, phoneme discrimination, phonological awareness, rapid automatized naming (RAN), phonological short-term memory	Phonological processing skills (linked to reading ability)	Children with SRD and SRD + SLI showed significant deficits in phonological skills. SRD + SLI group showed broader and more severe impairments, including phonological memory.
7	McCarthy et al. (2012)	USA	Ν = 129, Ages 9–10	SLI only, DD only, SLI + DD, TD	TOLD-P:2 (language in kindergarten), Woodcock Reading Mastery Test-Revised (Word Identification, Word Attack), language composites	Word reading accuracy (real and nonwords)	Children with DD and those with comorbid SLI/DD exhibited significant deficits in word reading accuracy (both real words and nonwords), whereas children with SLI only demonstrated word reading skills comparable to TD peers. Furthermore, word reading ability was a stronger predictor of spelling performance than oral language skills.
8	Kim and Lombardino (2013)	USA	*N* = 91, Ages 8–12	SRD, LIRD	GORT-IV (fluency & comprehension), CTOPP – RAN (naming speed)	Reading fluency, comprehension, naming speed	LIRD group had lower comprehension and verbal ability compared to SRD. Naming speed predicted fluency in both groups; verbal ability predicted comprehension only in LIRD.
9	Wong et al. (2015)	Hong Kong	*N* = 94, Ages 6.7–7.11	DD-only, SLI-only, DD + SLI, TD	HKCOLAS (oral language), HKT-P [II] (reading composite), RAN, phonological awareness, phonological memory	Word reading accuracy, speed, dictation	Children with DD (with or without SLI) performed significantly lower in reading accuracy and fluency compared to typically developing and SLI-only peers. Reading deficits were strongly associated with dyslexia, not SLI alone.
10	Loucas et al. (2016)	United Kingdom	*N* = 64, Ages 5–17	SLI only, SLI + RDI, No SLI/No RDI	CELF-3/4 (Receptive, Expressive, Total Language), TOWRE (Sight Word & Phonemic Decoding Efficiency), CNRep, CTOPP, NWD (experimental), WISC-III (PIQ)	Word and nonword reading, phonological awareness, phonological memory, nonword discrimination	Children with co-occurring SLI + RDI showed the poorest reading outcomes, particularly in nonword decoding and phonological processing. The SLI-only group performed comparably to typical peers.
11	Wong et al. (2017)	Hong Kong	Ν = 82, Ages 7.8–9.5	TD, SLI-only, DD-only, SLI + DD	HKT-P[II] (Chinese Word Reading), CLA-P (Reading Comprehension), HKCOLAS (Oral Language), Listening Span (Working Memory), experimental tasks (Comprehension Monitoring, Inferencing)	Word reading, reading comprehension	Children with co-occurring SLI + DD had the lowest reading comprehension. SLI-only and DD-only groups also performed below TD. Higher-level language skills and working memory uniquely predicted comprehension.
12	Mengisidou and Marshall (2019)	Greece	*N* = 149, Ages 8–12	DDLD, TD	L’Alouette Reading Test (Greek), Reading Test Alpha, Test of Detection and Investigation of Reading Difficulties (Porpodas, 2007)	Accuracy, fluency, and speed in word, pseudoword, and connected-text reading	Children with DDLD showed significantly lower performance across all reading measures compared to TD peers. Reading skills predicted phonological fluency beyond age and nonverbal ability.
13	Snowling et al. (2019)	UK	Ν = 234, Ages 3.5–8	TD, FR only (family risk without early language impairment), LI only (early language impairment without family risk), FR + LI	SWRT, WIAT-II (Spelling), YARC (Reading Comprehension), RAN	Word reading, spelling, comprehension	Children with both early Language Impairment (LI) and family risk showed the lowest reading performance at age 8. Early LI alone also predicted poorer reading outcomes, especially when combined with family risk for dyslexia.
14	Spanoudis et al. (2019)	Cyprus	*N* = 52, Ages 7–11	RD, SLI, COM, Control	Word Identification (WID), Word Attack (WAT), Word Chains (silent reading), CBM-Maze (maze reading comprehension), Test-A (reading comprehension)	Word reading accuracy and fluency, pseudoword reading, silent reading fluency, reading comprehension	COM group showed lowest performance across all reading measures. RD group had deficits mainly in decoding; SLI group showed difficulties primarily in comprehension.
15	Mengisidou et al. (2020)	Greece	Ν = 149, Ages 6–12	DDLD, TD	Alouette test, Reading Test Alpha (accuracy & fluency), Test of DIRD (syllable & non-word reading), multiple language tasks	Reading accuracy, fluency, non-word reading	DDLD group showed significantly lower reading fluency and non-word reading accuracy compared to TD; language and phonological skills predicted reading performance
16	Snowling et al. (2020)	UK	Ν = 234, Ages 8–9	DD, DLD, DD + DLD, TD	YARC (Reading Comprehension), SWRT, DTWRP, GNWRT, TOWRE (Word & Nonword Reading), CELF-4, TROG-II, ROWPVT	Word reading, Nonword reading, Reading comprehension	All clinical groups had poorer reading comprehension than controls. DD group showed mild difficulties (mainly in decoding), DLD group had significant comprehension problems (due to weak language), and the comorbid group had the most severe deficits.
17	Ehrhorn et al. (2021)	USA	*N* = 160, Ages 7–9	Dyslexia only, DLD only, DLD+Dyslexia, Typical	WRMT-3, CELF-4, CTOPP-2 Nonword Repetition (norm-referenced and experimental versions)	Word reading accuracy (real and pseudowords), reading fluency, nonword repetition	The DLD+Dyslexia group showed the most severe phonological processing deficits. Dyslexia-only and DLD-only groups had intermediate profiles. Phonological variability and audiovisual cues affected all groups similarly.
18	Duff et al. (2023)	USA	*N* = 448, Ages 7.5–9.5	D-only, DLD-only, DLD + D, TD	CELF-4 (language), WRMT-III (word reading), MAP Reading (school-based)	Word decoding, fluency, comprehension	Children with D and/or DLD had significantly lower reading achievement from 2nd to 4th grade compared to TD. The DLD + D group showed the most severe deficits. Growth rates were similar but gaps persisted over time.

## Discussion

The studies included in this systematic review consistently show that children diagnosed with both Developmental Dyslexia (DD) and Developmental Language Disorder (DLD) experience more severe and pervasive reading difficulties than those with only one of the two conditions. These difficulties extend across multiple domains, including decoding, reading comprehension, phonological fluency, and higher-level language processing.

With regard to the first research question, which concerned the core characteristics of reading ability in children with comorbid DD and DLD, Catts et al. (2005), Snowling et al. (2019), and Duff et al. (2023) consistently report that children with comorbid DD and DLD show the lowest performance across a wide range of reading measures. This disadvantage appears to persist over time, indicating long-term developmental challenges. The severity of these difficulties is attributed to the combined impact of phonological deficits and broader language weaknesses. According to Mengisidou et al. (2020) and McCarthy et al. (2012), phonological limitations undermine decoding efficiency, while deficits in semantics and syntax hinder reading comprehension. Similarly, the findings by Kim and Lombardino (2013) and Wong et al. (2017) highlight reduced reading speed, poor comprehension, and difficulties in linguistic integration among comorbid children.

Furthermore, the study by Ehrhorn et al. (2021) identified significant phonological processing deficits in children with comorbid DD and DLD, even beyond tasks directly related to reading. These findings support the notion that their reading difficulties stem from broader neurodevelopmental vulnerabilities as previous research has documented **(**Duff et al., 2023; Ehrhorn et al., 2021; Fraser et al., 2010; Wong et al., 2015). This perspective underscores the importance of comprehensive, multidimensional assessment grounded in the use of specific diagnostic tools and clinical markers to delineate reading and language profiles in comorbidity. Measures of academic achievement and reading, such as the WIAT-IV, together with decoding-focused assessments (e.g., TOWRE), support the identification of core difficulties associated with dyslexia, whereas language assessments (e.g., CELF) and clinical markers such as nonword repetition (NWR) are particularly informative for identifying Developmental Language Disorder. Profiling students across these domains supports differential diagnosis and facilitates alignment with evidence-based interventions, including structured literacy approaches for dyslexia and language-comprehension–focused interventions for DLD, particularly in cases of comorbidity.

Turning to the second research question, regarding the shared and distinct features of reading performance among children with DD, DLD, and the comorbid condition, research has shown that these three groups exhibit both overlapping difficulties and unique patterns. Specifically, all groups demonstrate difficulties in reading compared to typically developing peers; however, the nature and severity of these difficulties vary considerably. Children with DD tend to exhibit pronounced phonological deficits that impact decoding, word recognition, and reading fluency, while their reading comprehension is often relatively preserved (Fraser et al., 2010; Scuccimarra et al., 2008). In contrast, children with DLD primarily present impairments in semantic and syntactic processing, which adversely affect text comprehension, even in the absence of overt phonological difficulties (McArthur & Castles, 2011; Loucas et al., 2016). Notably, Scuccimarra et al. (2008) reported that dyslexic children with and without co-occurring language impairment showed similarly poor decoding performance in the early school years, suggesting that language impairment may not further compromise word-level decoding at initial stages of reading development. This finding highlights the importance of developmental timing, as the additive impact of comorbid language difficulties may become more pronounced later, when reading demands increasingly shift from basic decoding to fluency and comprehension.

Children with comorbid DD and DLD appear to combine the deficits of both diagnostic categories, resulting in the most compromised reading profiles. The studies by Eisenmajer et al. (2005), Spanoudis et al. (2019), and Wong et al. (2010) report that comorbid children show the lowest performance across almost all reading and comprehension measures. The co-occurrence of phonological and broader language impairments creates a cumulative burden that distinguishes them from single-disorder groups.

Within the current literature, comorbidity between Developmental Dyslexia and Developmental Language Disorder is increasingly conceptualized as the co-occurrence of two distinct neurodevelopmental disorders rather than as a qualitatively different condition or a simple severity continuum. Consistent with the Fully Distinct Hypothesis, the findings of the present review indicate that the increased severity observed in comorbid DD + DLD profiles is best explained by the combined impact of phonological deficits associated with dyslexia and broader oral language impairments characteristic of DLD, rather than by the presence of a new unified disorder or simply a more severe manifestation of a single difficulty. This interpretation aligns with contemporary perspectives in the field, which emphasize partially independent underlying cognitive and linguistic mechanisms contributing to reading and language outcomes.

Evidence suggests that DD has a stronger impact on decoding skills, while DLD more strongly affects language comprehension. When co-occurring, comorbidity is consistently associated with more extensive and severe learning difficulties compared to single-disorder profiles; however, the nature and mechanisms underlying this combined impact remain insufficiently understood and cannot yet be characterized as additive or multiplicative (Duff et al., 2023). Moreover, the existing literature has primarily focused on describing academic and cognitive characteristics, while comparatively little attention has been paid to the types of interventions and educational services these students receive, as well as to issues of mis- or under-identification of comorbidity due to diagnostic and educational/administrative classification practices. These findings highlight the need for cautious interpretation of severity patterns and for further research examining diagnostic processes and intervention pathways in children with comorbid profiles.

Finally, in relation to the third research question, which concerned the linguistic and cognitive predictors of reading performance, beyond core reading difficulties, research has highlighted several linguistic and cognitive factors associated with reading performance in children with comorbid developmental dyslexia (DD) and developmental language disorder (DLD). The synthesis of findings highlights phonological processing as a key predictor of reading outcomes in this population. Specifically, phonological awareness, phoneme discrimination, rapid automatized naming (RAN), and phonological short-term memory have been consistently linked to reduced decoding accuracy and reading fluency (Ehrhorn et al., 2021; McArthur & Castles, 2011; Mengisidou et al., 2020). In addition, phonological memory as indexed by nonword repetition (NWR) has been shown to be associated with decoding difficulties in children with dyslexia within comorbid DD + DLD groups (Catts et al., 2005; Hancock et al., 2025). In parallel, language comprehension, including semantic processing and syntactic organization, has emerged as a strong determinant of reading comprehension abilities (Snowling et al., 2020; Wong et al., 2017).

Several studies (e.g., Catts et al., 2005; Loucas et al., 2016) suggest that phonological proficiency may serve as a differentiating factor between children with DLD-only and those with DD or comorbid profiles, whereas broader language weaknesses primarily affect comprehension. The combined presence of phonological and language deficits leads to reduced reading fluency, limited lexical access, and inefficient reading resulting from insufficient consolidation and integration of orthographic, phonological, and semantic representations, rather than from ineffective use of reading strategies (Ehri, 2014) and poor deployment of reading strategies. Overall, children with comorbid DD and DLD exhibit overlapping impairments in core cognitive and linguistic mechanisms that are critical to reading development. Understanding these predictive factors is essential for improving early identification practices and designing personalized interventions tailored to complex profiles.

Taken together, the findings from the studies included in this systematic review in answer to the three research questions provide converging evidence that comorbidity of DD and DLD is associated with more complex and severe reading difficulties compared to single-disorder profiles, without implying a qualitatively distinct condition or a clearly additive effect. Across decoding, comprehension, and cognitive predictors, children with comorbid diagnoses consistently show more severe, pervasive, and persistent reading difficulties than those with single disorders. These patterns raise important theoretical and practical considerations regarding the nature of comorbidity, the mechanisms underlying reading failure, and the adequacy of current diagnostic and intervention models. The consistent co-occurrence of phonological and language deficits highlights the need for careful empirical documentation of comorbidity, given their combined impact on both decoding and comprehension processes. This pattern, evident in the studies by Catts et al. (2005), Snowling et al. (2019), and Mengisidou et al. (2020), underscores the need for early detection and accurate differential diagnosis, in order to provide more effective and individualized educational support.

Despite the importance οf these insights, several limitations of the present review must be acknowledged. A number of the included studies relied on small or unbalanced samples, particularly in comorbid groups, which reduces statistical power and limits the generalizability of the findings. For example, in the study by Catts et al. (2005) the final subgroup of children with comorbidity was relatively small compared to the other groups, while in Wong et al. (2010) the group sizes differed substantially, leading to unbalanced samples. In addition, the majority of the studies employed cross-sectional designs, which restricts understanding of the developmental trajectory of reading difficulties, whereas longitudinal research remains scarce (e.g., Duff et al., 2023; Snowling et al., 2019). A further limitation concerns the considerable variability in the assessment tools used for reading and language, making comparisons and synthesis of findings across studies difficult. For instance, Mengisidou et al. (2020) relied on Greek instruments such as the Alouette and the Test of DIRD, whereas Spanoudis et al. (2019) employed different measures, such as the CBM-Maze and Test-A for comprehension, highlighting methodological heterogeneity. An additional limitation concerns variability in nonverbal IQ (NVIQ) criteria across the included studies, as primary studies adopted different diagnostic thresholds, which may make comparisons across findings more difficult. Finally, the fact that the majority of studies conducted with English-speaking populations restricts the generalizability of the conclusions to languages with different phonological and morphological characteristics that shape reading development. Although the present review also included studies with Greek-speaking (Mengisidou & Marshall, 2019; Mengisidou et al., 2020; Spanoudis et al., 2019, 2019), Chinese-speaking (Wong et al., 2010, 2015, 2017), and Italian-speaking populations (Scuccimarra et al., 2008; Brizzolara et al., 2006), their number remains limited compared to English-speaking samples.

Methodological limitations related to the review process itself should also be considered. A further limitation concerns the restriction of the search to studies published between 2000 and 2023. While this decision was made to focus on more recent research and current conceptualizations of the relationship between language and reading, it excludes earlier seminal work, particularly longitudinal and epidemiological studies, on the comorbidity between language and reading difficulties, which has provided important insights into their developmental relationship. The systematic examination of this earlier literature falls outside the scope of the present review. Future research could usefully re-examine this body of work, as this would provide a more comprehensive and historically grounded understanding of the comorbidity between language and reading difficulties. In addition, methodological variability across studies limited the possibility of conducting a quantitative synthesis. A meta-analysis was not conducted due to the heterogeneity of study samples, assessment targets, and outcome measures. Nevertheless, the search and selection process was rigorously predefined and carried out with methodological consistency. Additionally, it should be noted that the study selection and appraisal process was conducted by a single reviewer. Although the search strategy and the inclusion and exclusion criteria were clearly predefined and consistently applied, the absence of a second independent reviewer may introduce potential selection bias and limits the assessment of interrater reliability.

Finally, the findings of this review carry important implications for both research and practice. Children with comorbid DLD and DD present a unique profile of reading and language difficulties, which may not be fully captured by current assessment practices. These findings highlight the importance of clearly recognizing the co-occurrence of DLD and DD within existing diagnostic frameworks, rather than implying the need for a separate diagnostic category or label. In addition, the findings underscore the relevance of considering both linguistic and reading-related domains when interpreting comorbid profiles. In terms of educational planning, greater awareness and systematic identification of comorbid profiles may support more appropriate educational planning and inclusive teaching practices, without implying specific intervention mandates. Future research should prioritize longitudinal studies, expand beyond English-speaking populations, and adopt more consistent and comparable methodological frameworks to improve generalizability and theoretical integration.

## Supplementary Information

Below is the link to the electronic supplementary material.

Supplementary Material 1

## Data Availability

Data will be made available on request.
